# Luxation radio carpienne ouverte pure: à propos d'un cas

**DOI:** 10.11604/pamj.2016.23.23.8622

**Published:** 2016-02-01

**Authors:** Adil El Alaoui, Mouhcine Sbiyaa

**Affiliations:** 1 Service de Chirurgie Traumato-Orthopédique (A), CHU Hassan II, Fès, Maroc

**Keywords:** luxation, pure, radiocarpienne, Dislocation, pure, radio carpal

## Image en médecine

La luxation radio carpienne est rare, sa fréquence est de 0,2% des luxations. La forme pure reste une entité exceptionnelle. Nous rapportons une observation typique de cette lésion. Il s'agit d'un patient âgé de 32 ans, victime d'un accident de la voie public. Admis aux urgences ou un examen du membre supérieur gauche a objectivé une plaie transversale en regard de la face palmaire du poignet gauche (A), l'examen vasculo-nerveux est sans particularités. Une radiographie du poignet gauche face et profil a mis en évidence une luxation postérieure pure radio carpienne gauche (B). Le patient a été opéré 2 heures après le traumatisme au bloc opératoire, sous anesthésie locorégionale. Après la réduction de la luxation radio carpienne le patient a bénéficié d'un embrochage radio-lunaire et radio-scaphoidienne puis un embrochage transversale radio-lunaire. La radiographie de contrôle a confirmé la réduction de la luxation (C, D). Le patient a bénéficié d'une immobilisation du poignet pendant 6 semaines, suivie d'une ablation de broches (E) puis des séances de rééducation. Après un recul de 18 mois, l’évolution était bien, avec une mobilité normale du poignet.

**Figure 1:**
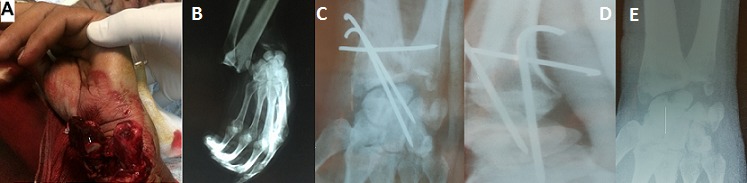
(A) image montrant une luxation ouverte radio carpienne; (B) radiographie du poignet gauche montrant une luxation pure postérieure radio carpienne; (C) radiographie de contrôle du poignet face après réduction de la luxation et stabilisation du poignet par embrochage; (D) radiographie du poignet de profil après réduction de la luxation et stabilisation du poignet par embrochage; (E) radiographie du poignet gauche après ablation des broches

